# Endovascular Intervention and the Vascular Glycocalyx in Patients with Chronic Limb-Threatening Ischaemia: A Prospective Observational Study

**DOI:** 10.3390/ijms27136011

**Published:** 2026-07-04

**Authors:** Aleksander Truszyński, Urszula Jakobsche-Policht, Andrzej Szuba

**Affiliations:** Department of Angiology and Internal Medicine, Faculty of Medicine, Wroclaw Medical University, 50-556 Wroclaw, Poland; urszula.jakobsche-policht@umw.edu.pl (U.J.-P.); andrzej.szuba@umw.edu.pl (A.S.)

**Keywords:** chronic limb-threatening ischaemia, glycocalyx, endothelium, endovascular, PTA

## Abstract

This single-center prospective observational study evaluated peri-procedural changes in circulating markers of endothelial glycocalyx injury in patients with chronic limb-threatening ischemia undergoing lower extremity endovascular treatment. Fifty patients (mean age 72.2 years; 56% with diabetes mellitus; 42% current smokers) underwent percutaneous transluminal angioplasty, most commonly involving the femoropopliteal segment and using 4F introducer sheaths. Serum concentrations of syndecan-1, heparan sulfate, and hyaluronic acid were measured using ELISA before and one day after the procedure. No significant differences were observed between baseline and post-procedural biomarker levels, with minimal median changes, overlapping interquartile ranges, and negligible paired effect sizes. Although individual biomarker trajectories demonstrated considerable interindividual variability, no consistent directional trend was identified. Furthermore, demographic and clinical factors, including age, sex, diabetes, and smoking status, as well as procedural variables such as stent implantation, sheath size, and intervention level, were not associated with biomarker changes in univariate or multivariable analyses. These findings suggest that contemporary endovascular revascularization is biologically well tolerated and does not induce detectable systemic endothelial glycocalyx disruption. Nevertheless, the interpretation of these findings is limited by the moderate sample size and single-center design of the study. In addition, syndecan-1, heparan sulfate, and hyaluronic acid are not exclusive markers of endothelial glycocalyx injury and may also reflect extracellular matrix turnover and tissue remodeling.

## 1. Introduction

Lower extremity arterial disease (LEAD) is a manifestation of peripheral artery disease (PAD), a chronic condition related to atherosclerosis that affects over 200 million people worldwide [1,2,3]. It poses a significant problem at both the individual and societal levels. It is the third leading cause of cardiovascular mortality, following heart attack and stroke. LEAD may be asymptomatic or cause a wide range of symptoms. In mild cases, the main symptom is intermittent claudication, which causes calf pain with walking that subsides with rest. In its most severe forms, LEAD leads to chronic limb-threatening ischemia (CLTI), which includes rest pain and the presence of ulcers or necrosis. It often leads to the need for limb amputation.

An intact endothelial structure is essential for the proper functioning of arteries [4]. An integral part of the endothelium is the glycocalyx, a carbohydrate-rich layer facing the vessel’s interior. Sometimes described as a pericellular matrix, the glycocalyx is a polysaccharide structure that protrudes from the cell body and forms an essential component of the extracellular environment [5,6]. Its primary constituents, such as syndecan-1, heparan sulfate (HS), and hyaluronan (HA), are not only structural anchors for this luminal layer but are also fundamental components of the extracellular matrix (ECM) found ubiquitously throughout various tissues [7,8,9]. Consequently, these molecules play a dual role, maintaining both the structural integrity of the endothelial surface layer and the broader homeostatic and structural functions of the systemic extracellular environment [7,10]. The glycocalyx provides anti-coagulant and anti-adhesive effects on the surface of endothelial cells. Moreover, it can shield endothelial cells from oxidative stress. Damage to the endothelium is a key factor in the development of atherosclerotic lesions, as it impairs vasodilation, promotes arterial wall proliferation, and induces a prothrombotic state [11].

To date, numerous diagnostic methods have been developed to diagnose peripheral artery disease, including those that consider endothelial dysfunction. The former include the ankle–brachial index (ABI), toe–brachial index (TBI), and measurement of Transcutaneous Oxygen Partial Pressure (TcPO2) [12,13]. The latter comprises flow-mediated dilation (FMD), reactive hyperemia index (RHI), and arterial pulse waveform analysis (aPWA) [14,15].

While classical hemodynamic parameters such as the ABI and TBI are used to confirm macrovascular flow-limiting lesions, they often fail to capture the early functional changes occurring within the vascular wall. Endothelial dysfunction, marked by the degradation of the endothelial glycocalyx, is a precursor to macrovascular plaque progression [16,17]. Research has shown that major vascular surgeries involving global or regional ischemia—such as aortic aneurysm repair—trigger massive systemic shedding of glycocalyx constituents into the circulation. In contrast, non-invasive approaches such as exercise rehabilitation have been shown to affect vascular and inflammatory biomarkers, potentially reflecting adaptive microcirculatory responses. Understanding the biological impact of contemporary endovascular procedures on these delicate structures is essential to optimizing clinical outcomes and minimizing systemic vascular trauma [18,19].

Proper endothelial function contributes to slowing the progression of atherosclerotic changes and promotes faster recovery of peripheral circulation after exercise-induced temporary ischemia [18].

Several studies suggest that damage to the vascular endothelial glycocalyx can be assessed by measuring circulating biomarkers. These markers include: syndecan-1, heparan sulfate (HS), and hyaluronic acid (HA) [20,21,22].

The role of vascular endothelial glycocalyx damage in the development of atherosclerosis and, consequently, peripheral artery disease has been studied extensively [23,24,25]. However, little is known about the effects of endovascular revascularization on the integrity, damage, and regeneration of the glycocalyx. We hypothesize that endovascular revascularization, although performed locally, may induce a systemic increase in circulating markers of glycocalyx damage due to direct mechanical injury to the vascular endothelium and its glycocalyx layer. During the procedure, manipulation of guidewires and catheters within the vessel lumen, balloon inflation, and stent deployment may contribute to disruption of the endothelium and glycocalyx, resulting in shedding of glycocalyx components into the circulation. This knowledge gap underscores the need for further investigation and may provide insights into novel therapeutic and monitoring strategies.

In this study, we evaluated the impact of angioplasty on endothelial glycocalyx injury by measuring circulating concentrations of glycocalyx biomarkers in patients with chronic limb-threatening ischemia (CLTI). Our primary aim was to determine whether endovascular treatment is associated with systemic glycocalyx injury and to identify potential predictors of glycocalyx damage, thereby providing a foundation for future research and clinical applications.

## 2. Results

### 2.1. Baseline Characteristics

Baseline characteristics are presented in Table 1. The cohort comprised 50 patients (mean age 72.20 years), with diabetes and current smoking present in 56% and 42% of cases, respectively. Most procedures were performed using 4F introducer sheaths (80%) and targeted the femoropopliteal segment (56%), with fewer below-the-knee or multilevel interventions.

### 2.2. Changes in Endothelial Glycocalyx Injury Markers

**Figure 1:**
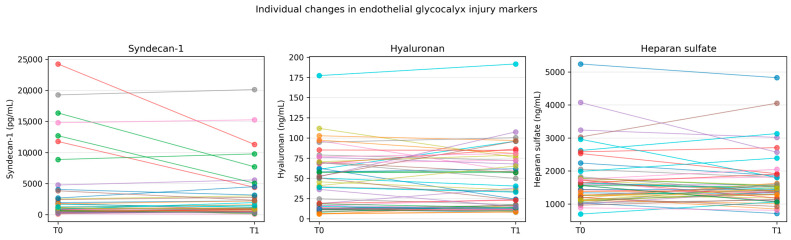
Individual trajectories of endothelial glycocalyx injury marker concentrations before and after endovascular intervention. Paired line (“spaghetti”) plots present absolute concentrations of Syndecan-1, hyaluronan, and heparan sulfate measured at baseline (T0, pre-procedural) and on the first day after the procedure (T1, post-procedural). Each line represents one patient.

Changes in endothelial glycocalyx injury markers are summarized in Table 2. No statistically significant differences were observed between baseline and post-procedural concentrations of Syndecan-1, hyaluronan, or heparan sulfate. Median changes were small with overlapping interquartile ranges, and effect sizes for paired comparisons were uniformly negligible, indicating minimal within-patient variation following the intervention. Individual patient trajectories are illustrated in Figure 1 (and Supplementary Figure S1 for logarithmic scaling), demonstrating considerable interindividual variability without a consistent directional trend.

### 2.3. Predictors of Biomarker Changes

Univariate analyses exploring potential clinical and procedural predictors of biomarker changes are presented in Table 3. No significant associations were identified between demographic characteristics or comorbidities, including age, sex, diabetes, and smoking status, and changes in Syndecan-1, hyaluronan, or heparan sulfate concentrations. Procedural factors, such as stent implantation, introducer sheath size, and intervention level, were likewise not associated with biomarker changes.

Multivariable linear regression models are summarized in Supplementary Tables S1 and S2. In the primary model adjusted for age, diabetes mellitus, and introducer sheath size, no variables were independently associated with post-procedural changes in any biomarker. Inclusion of smoking status in an extended model yielded consistent results, with no significant independent predictors identified.

## 3. Discussion

The study aimed to assess damage to the glycocalyx layer following endovascular intervention by measuring circulating biomarkers. This is the first study to evaluate the direct endothelial injury during percutaneous intervention in patients with CLTI by measuring circulating biomarker concentrations.

We did not observe significant acute increases in circulating markers of endothelial glycocalyx injury. Concentrations of syndecan-1, hyaluronan, and heparan sulfate remained largely unchanged after the procedure, with small median differences and uniformly negligible effect sizes. Furthermore, neither clinical characteristics nor procedural factors were independently associated with biomarker changes.

The absence of significant biomarker elevation may indicate that endovascular treatment is associated with limited systemic endothelial injury. Although mechanical manipulation of the vessel wall is unavoidable, the magnitude of glycocalyx shedding appears minimal at the systemic level, suggesting that the biological impact of these procedures may reflect predominantly local rather than systemic effects.

It is essential to interpret our findings within the context of the ‘dual effect’ of these biomarkers on vascular biology. While syndecan-1, HS, and HA are established indicators of endothelial glycocalyx shedding, they are also integral constituents of the ECM. Hyaluronan is a major natural component of the ECM in all tissues, crucial for water homeostasis and cellular metabolism [7,8]. Similarly, heparan sulfate is ubiquitously expressed both on cell surfaces and within the ECM as free fragments. In cardiovascular pathology, syndecan-1 additionally acts as a modulator of fibrosis within the myocardial and vascular ECM [9]. Therefore, the elevated baseline values in our study population (mean age 72, 56% diabetes) likely reflect not only chronic luminal glycocalyx degradation but also a broader state of chronic ECM remodeling and panvascular aging [26]. This distinction is critical, as these shed components can act as damage-associated molecular patterns (DAMPs), entering the circulation to propagate inflammation and endothelial activation at distant sites, creating a vicious cycle of systemic vascular vulnerability [26,27].

To date, many researchers have conducted numerous studies and analyses on the glycocalyx, familiarizing the scientific community, clinicians, and researchers with issues related to its structure, physiology, and pathophysiology, as well as diagnostic methods, and setting further directions for research. Starting with the isolation of the glycocalyx as a separate structure of the vessel wall, through the identification of the molecules that comprise it, to the determination of the mechanisms that influence damage to its structure [16,28,29].

The glycocalyx serves a fundamental protective role for the endothelium and is indispensable for normal vascular function. It operates as an interface between circulating substances and endothelial cells, regulates vascular permeability, and acts as a mechanosensor of hemodynamic shear stress [30].

One available method for detecting glycocalyx is the ELISA test, which can detect its components, including Syndecan-1, HA, and HS, as used in our study [16,31].

To the best of our knowledge, this is the first study to evaluate the impact of endovascular treatment on damage to the vascular endothelial glycocalyx in patients with CLTI. The available literature comprises studies that evaluated glycocalyx biomarker concentrations after interventions in other vascular beds and in the context of clinical symptoms. Liang D. et al. measured serum levels of syndecan-1, HA, and HS in patients who underwent large-vessel occlusion revascularization for ischemic stroke. Their study showed an increase in HA and HS concentrations immediately after patency restoration (0 h), followed by fluctuations. In contrast, the syndecan-1 level dropped rapidly after reperfusion and peaked again 36 h later. The differences were not statistically significant [32]. Another study by Momot et al. aimed to compare two transradial approaches with respect to clinical and biochemical outcomes; however, it assessed differences in radial artery access type and biomarker levels. Nevertheless, no statistically significant changes in biomarker concentration have been noted [33].

In the available literature, plasma values for the endothelial glycocalyx biomarkers included in our study have been reported in healthy reference populations. Regarding syndecan-1, Tavalaie et al. reported a mean serum level of 15.20 ± 4.36 ng/mL in healthy controls, while Çekiç et al. observed a mean concentration of 34.1 ± 8.0 ng/mL [34,35]. For HA Cylwik et al. determined a median serum concentration of 13.7 ng/mL (IQR: 5.8–19.7 ng/mL) in healthy subjects [7]. Finally, HS levels in healthy cohorts have been reported by Koźma et al. as a mean plasma concentration of 1.35 ± 0.18 µg/mL, whereas Sabol et al. found median free HS levels ranging from 0.15 µg/mL to 0.21 µg/mL depending on menopausal status [8,36]. A comparison of these reference values with the baseline results from our study population suggests that our patients exhibit significant glycocalyx injury.

Despite searching numerous scientific repositories, we have not found any studies on glycocalyx damage during endovascular interventions in patients with ischemic heart disease. Some studies suggest a significant increase in biomarker concentrations (Syndecan-1, HA, HS) in patients undergoing CABG and in cases of postcardiac arrest syndrome, where ischemia and the ischemia/reperfusion mechanism are thought to explain this phenomenon [37,38]. However, because endovascular methods were not included in the research methodology, we can only assume that contemporary endovascular techniques may minimize systemic endothelial trauma.

A meta-analysis by Kaczmarczyk et al. assessed the impact of endovascular treatment on endothelial function [39]. 8 studies were included with 466 patients enrolled across all studies [40,41,42,43,44,45,46,47]. The results of this review were inconclusive, indicating a possible positive effect of PTA on endothelial function. The studies included in this meta-analysis do not directly address the glycocalyx; however, as mentioned in the introduction, the glycocalyx is an integral part of the endothelium and thus an intact glycocalyx is a prerequisite for proper endothelial function. Furthermore, we presume that PTA’s effect on endothelial function is more complex than a single local interaction with the glycocalyx.

Collectively, our findings suggest that the absence of significant acute elevations in glycocalyx biomarkers in our cohort indicates that contemporary endovascular techniques may induce less systemic endothelial and inflammatory stress than traditional surgical revascularization. In the available literature, major open vascular surgeries involving global or regional ischemia (such as aortic aneurysm repair or cardiopulmonary bypass) are documented to trigger dramatic, multifold increases in circulating markers. For instance, Rehm et al. reported a 42- to 65-fold increase in syndecan-1 and a 10- to 19-fold increase in heparan sulfate during the early reperfusion phase [38]. These massive spikes are attributed to intense ischemia/reperfusion injury, a primary driver of glycocalyx degradation, which was likely minimized by the localized, less invasive nature of our endovascular approach [38]. Even non-invasive stressors, such as acute exercise in PAD patients, have been shown to cause transient functional deterioration of distal arteries and increases in inflammatory adhesion molecules, further highlighting the biological stability observed in our results [18]. The clinical implications require further investigation. We are currently developing a follow-up programme for patients after PTA treatment to assess glycocalyx marker concentrations and the occurrence of restenosis and reocclusion.

Several explanations may account for the observed stability of glycocalyx markers. First, endovascular procedures are generally less invasive than open surgical revascularization and may therefore induce less systemic inflammatory and endothelial stress. Second, potential endothelial injury may remain predominantly localized to the treated arterial segment and not be reflected in circulating biomarkers. Third, transient biomarker fluctuations might have occurred outside the sampling window and thus remained undetected, as observed in the study by Liang D. et al. [32].

## 4. Materials and Methods

### 4.1. Study Design

A single-center, prospective, observational cohort study was conducted between February 2024 and August 2024 in inpatient settings at the Wroclaw University Clinical Hospital in Poland. All patients enrolled in the study were hospitalized for CLTI and underwent percutaneous transluminal angioplasty (PTA).

### 4.2. Inclusion Criteria/Eligibility

Patients over 50 years of age, both women and men, who were eligible for PTA due to chronic limb-threatening ischemia. Written consent was obtained from eligible individuals. CLTI was defined according to SVS reporting standards as rest pain or the presence of non-healing ulcers or necrosis in the lower limbs (Rutherford classification 4–6) attributable to confirmed PAD [48].

The vessels affected by atherosclerosis that caused symptoms in patients had to be located below the common femoral artery.

The study participants were divided into subgroups based on whether the procedure involved vessels above the knee (ATK), below the knee (BTK), or multilevel.

### 4.3. Exclusion Criteria

The patients were excluded from the study in case of unsuccessful procedure, defined as more than 50% residual stenosis confirmed angiographically or by duplex ultrasound, on the day after the procedure, acute myocardial ischemia, stroke, respiratory failure, uncontrolled hypertension, decompensated congestive heart failure, or end-stage kidney disease.

### 4.4. Data Collection

Test samples were collected pre- (T0) and postintervention (T1), where T0 refers to the day before and T1 to the day after the procedure. The day after the procedure, patients were examined in the ultrasound room, where post-procedure vessel patency was assessed.

Quantitative assessment of glycocalyx damage markers—syndecan-1, heparan sulfate, hyaluronic acid—was performed using enzyme-linked immunosorbent assay kits (Human SDC1, biorbyt, Cambridge, UK; Human SA, biorbyt, Cambridge, UK; Human HA, biorbyt, Cambridge, UK) intended for scientific research. Venous blood was used for the experiment and, after clotting, was centrifuged (MPW M-SCIENCE, MPW MED.INSTRUMENTS, Warsaw, Poland) at room temperature at 1000× *g* for 15 min and stored at −80 °C. Before the experiment, the samples were gently thawed. The ELISA test was performed according to the manufacturer’s guidelines, using plates coated with anti-SDC-1, anti-SA, and anti-HA monoclonal antibodies, respectively, and following the incubation and washing conditions. Absorbance values were determined photometrically (Multiscan FC, Thermo Fisher Scientific, Massachusetts, GA, USA) at 450 nm.

### 4.5. Power Calculation

Based on a study conducted by Nieuwdorp et al. [49], 10 participants were required in a longitudinal study to detect a 0.2 µm change in glycocalyx thickness with 80% power and a two-sided α level of 0.05. The present study included 50 patients, exceeding this minimum requirement.

### 4.6. Statistical Analysis

Statistical analyses were performed using JASP (v0.18.3; University of Amsterdam, the Netherlands) and Python (v3.12.1; Python Software Foundation, Wilmington, DE, USA) to enable reproducible data processing and visualization. Continuous variables were assessed for distributional assumptions using histograms and Q–Q plots. Given the non-normal, skewed distribution of biomarker concentrations, nonparametric methods were used for group comparisons. Effect sizes were calculated as r = Z/√n, where Z is the standardized test statistic obtained from the Wilcoxon test and n is the number of paired observations. The magnitude of the effect size was interpreted according to Cohen’s criteria, with values of 0.1, 0.3, and 0.5 corresponding to small, medium, and large effects, respectively [50]. All statistical tests were two-sided, and *p*-values < 0.05 were considered statistically significant. Univariate analyses were conducted to explore potential predictors of biomarker changes. Associations with continuous variables were assessed using Spearman’s rank correlation. Binary categorical variables were compared using the Mann–Whitney U test, whereas variables with more than two categories were analyzed using the Kruskal–Wallis test.

To identify factors associated with biomarker changes following endovascular intervention, separate multivariable linear regression models were constructed for each biomarker. The dependent variable was defined as the natural logarithm of the post- to pre-procedural biomarker ratio [ln(T1/T0)], where values greater than zero indicate an increase, values less than zero indicate a decrease, and a value of zero indicates no change [51]. Log transformation was applied to reduce skewness, improve model assumptions, and allow proportional rather than absolute changes to be analyzed.

Covariates were selected a priori based on clinical relevance and biological plausibility. Age and diabetes mellitus were included because of their known associations with endothelial dysfunction and glycocalyx injury, whereas introducer sheath size was included as a procedural variable that may reflect the extent of vascular manipulation. Smoking status was added in an extended model because of its established relationship with vascular injury and inflammation. Regression coefficients (β) represent the independent association of each covariate with biomarker change after adjustment for all other variables included in the model.

To minimize overfitting given the sample size, the number of predictors was deliberately restricted [52,53]. Regression results are reported as β coefficients with 95% confidence intervals and corresponding *p*-values. Binary variables were coded using dummy variables, with lower-risk categories used as references (no diabetes, non-smoking status, and 4F sheath).

## 5. Conclusions

From a clinical perspective, the absence of detectable systemic glycocalyx disruption may be reassuring, as preservation of endothelial integrity is increasingly recognized as an important determinant of vascular outcomes. These results suggest that modern endovascular revascularization is biologically well tolerated at the systemic level.

### Limitations

Several limitations should be acknowledged. The study was conducted at a single center with a moderate sample size, and biomarker measurements serve as indirect surrogates of endothelial injury. The power calculation presented in materials and methods section indicates that the group was sufficient for the primary analysis, nevertheless it may have limited the ability to detect more subtle associations in subgroup analyses. Moreover, only early post-procedural time points were assessed, which may not fully capture transient or delayed responses.

Notably, effect sizes for paired comparisons were uniformly small according to Cohen’s criteria. This finding supports the interpretation that the lack of statistical significance was not solely attributable to limited power, but rather reflects genuinely minimal biological changes following the intervention.

Although ELISA enables convenient quantitative assessment of glycocalyx-associated components, including heparan sulfate and hyaluronan, these molecules are not exclusive to the endothelial glycocalyx; they are also present on various cell surfaces and within the extracellular matrix. Therefore, ELISA measurements may be affected by contributions from non-endothelial compartments, which should be considered when interpreting the results [31]. However, in our study, we compared marker concentrations before and after the intervention, thereby eliminating the influence of other factors.

## Figures and Tables

**Table 1:** Baseline characteristics.

Variable		
N		50
Age, years		
	mean (SD)	72.70 (8.75)
	median (IQR)	71.50 (67.25–76.75)
Female, *n* (%)		21 (42.00)
Diabetes mellitus, *n* (%)		28 (56.00)
Smoking, *n* (%)		21 (42.00)
Stent implantation, *n* (%)		18 (36.00)
Sheath size, *n* (%)		
	4F	40 (80.00)
	6F	10 (20.00)
Intervention level, *n* (%)		
	ATK	28 (56.00%)
	BTK	15 (30.00%)
	Multilevel	7 (14.00%)

Continuous variables are presented as mean (standard deviation) and median (interquartile range). Categorical variables are presented as number (percentage). SD: standard deviation; IQR: interquartile range; ATK: involved vessels above the knee; BTK: involved vessels below the knee. Multilevel: involvement of both ATK and BTK segments.

**Table 2:** Changes in endothelial glycocalyx injury markers between baseline (T0) and post-procedural (T1) measurements.

Biomarker	T0	T1	T1–T0	*p* Value	Effect Size
Syndecan-1 (pg/mL)	888.10 (498.50–2298.30)	935.80 (531.10–2294.70)	68.60 (−267.20–365.50)	0.41	0.12
Hyaluronic acid (ng/mL)	43.30 (13.30–69.30)	34.80 (13.80–72.40)	−0.30 (−6.60–4.00)	0.63	0.070
Heparan sulfate (ng/mL)	1558.70 (1184.30–1779.00)	1406.60 (1205.40–1803.70)	−46.70 (−307.90–175.70)	0.31	0.14

Data are presented as median (interquartile range). Paired comparisons between T0 and T1 were performed using the Wilcoxon signed-rank test. Effect sizes were calculated as r = Z/√n and interpreted according to Cohen’s criteria. Concentrations are shown in original measurement units.

**Table 3:** Univariate predictors of change in endothelial glycocalyx injury markers.

Predictor	Δ Syndecan-1	Δ Hyaluronan	Δ Heparan Sulfate
Clinical predictors			
Age	0.86 a	0.86 a	0.30 a
Sex	0.80 b	0.69 b	0.99 b
Diabetes mellitus	0.73 b	0.35 b	0.84 b
Current smoking	0.38 b	0.25 b	0.29 b
Procedural predictors			
Stent implantation	0.99 b	0.47 b	0.060 b
Sheath size	0.71 b	0.51 b	0.57 b
Intervention level	0.98 c	0.81 c	0.13 c

Values represent *p* values from univariate analyses. Associations with continuous variables were assessed using Spearman’s rank correlation (a), and categorical variables using the Mann–Whitney U test (b) or the Kruskal–Wallis test (c). *p* values < 0.05 were considered statistically significant.

## Data Availability

The data presented in this study are available on request from the corresponding author.

## Supplementary Materials

The following supporting information can be downloaded at: https://www.mdpi.com/article/10.3390/ijms27136011/s1.
