# Supplementary material for: Endovascular Intervention and the Vascular Glycocalyx in Patients with Chronic Limb-Threatening Ischaemia: A Prospective Observational Study
Source: Int J Mol Sci. 2026 Jul 4;27(13):6011. doi: 10.3390/ijms27136011 (PMC13361676; doi:10.3390/ijms27136011)
Supplement: Supplementary file 1 [file ijms-27-06011-s001.zip › Suplementary Files.pdf]

Supplementary Figure S1. Individual trajectories of endothelial glycocalyx injury marker concentrations on a logarithmic scale.

CAPTION: Paired line plots display concentrations of Syndecan-1, hyaluronan, and heparan sulfate measured at baseline (T0, pre-procedural) and on the first day after the procedure (T1, post-procedural). The y-axis is presented on a logarithmic scale to improve visualization of proportional changes and reduce the influence of extreme values. Each line represents one patient.

Supplementary Table S1. Multivariable linear regression models for log-transformed biomarker ratios (Model A)

Outcome	Predictor	β (95% CI)	*p* value
Syndecan-1			
	Age	-0.00 (-0.03 to 0.02)	0.74
	Diabetes mellitus	0.21 (-0.21 to 0.63)	0.32
	Sheath size	-0.02 (-0.54 to 0.50)	0.94
Hyaluronan			
	Age	0.00 (-0.01 to 0.02)	0.64
	Diabetes mellitus	0.10 (-0.14 to 0.33)	0.41
	Sheath size	0.04 (-0.25 to 0.32)	0.81
Heparan sulfate			
	Age	-0.00 (-0.01 to 0.00)	0.30
	Diabetes mellitus	-0.01 (-0.15 to 0.13)	0.88
	Sheath size	0.05 (-0.13 to 0.22)	0.58

Separate linear regression models were fitted for each biomarker using log-transformed ratios [ln(T1/T0)]. **Model A:** Covariates included age, diabetes mellitus, and introducer sheath size. Reference categories were no diabetes and 4F sheath.

Supplementary Table S2. Multivariable linear regression models including smoking status (Model B)

Outcome	Predictor	β (95% CI)	*p* value
Syndecan-1			
	Age	-0.01 (-0.04 to 0.02)	0.38
	Diabetes mellitus	0.14 (-0.29 to 0.58)	0.51
	Current smoking	-0.29 (-0.80 to 0.23)	0.27
	Sheath size	-0.00 (-0.52 to 0.52)	0.99
Hyaluronan			
	Age	-0.00 (-0.02 to 0.01)	0.82

	Diabetes mellitus	0.06 (-0.18 to 0.30)	0.63
	Current smoking	-0.17 (-0.45 to 0.12)	0.25
	Sheath size	0.05 (-0.24 to 0.34)	0.74
Heparan sulfate			
	Age	-0.00 (-0.01 to 0.01)	0.46
	Diabetes mellitus	-0.01 (-0.15 to 0.14)	0.94
	Current smoking	0.02 (-0.15 to 0.19)	0.82
	Sheath size	0.05 (-0.13 to 0.22)	0.60

Separate linear regression models were fitted for each biomarker using log-transformed ratios [ln(T1/T0)]. **Model B:** Covariates included age, diabetes mellitus, smoking status, and introducer sheath size. Reference categories were no diabetes, non-smoking status, and 4F sheath.
