# Supplementary figures and images for: Endovascular Intervention and the Vascular Glycocalyx in Patients with Chronic Limb-Threatening Ischaemia: A Prospective Observational Study
Source: Int J Mol Sci. 2026 Jul 4;27(13):6011. doi: 10.3390/ijms27136011 (PMC13361676; doi:10.3390/ijms27136011)

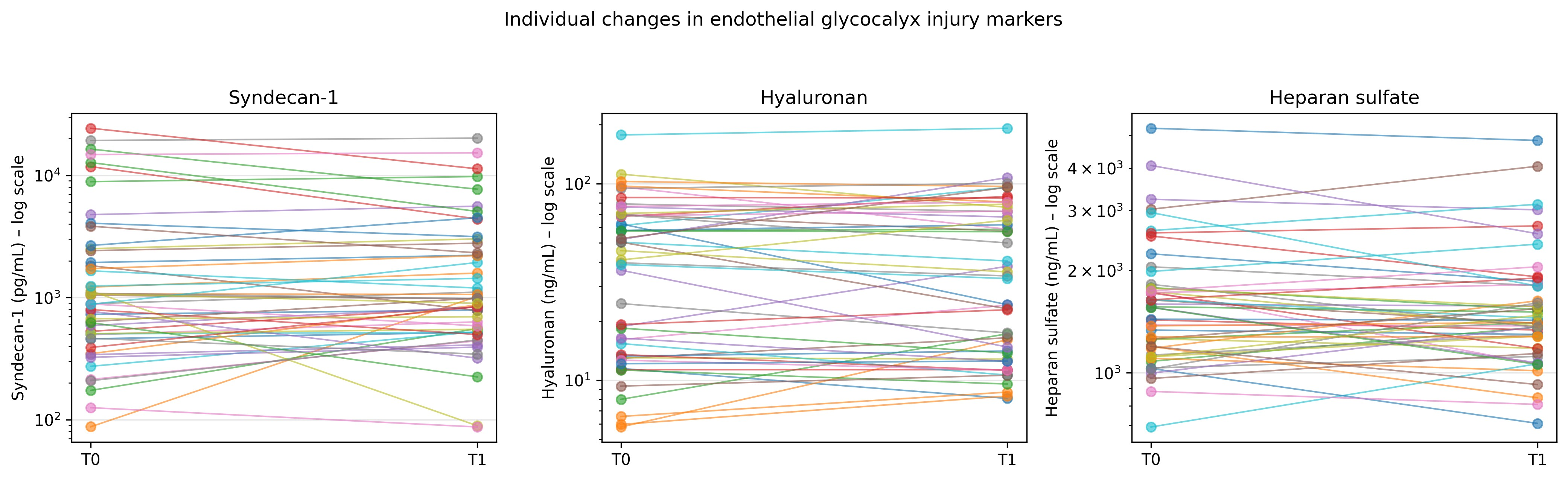

Supplement: Supplementary file 1 [file ijms-27-06011-s001.zip › Fig_S1.tif]
